# Identification and Functional Analysis of miRNAs in Extracellular Vesicles of Semen Plasma from High- and Low-Fertility Boars

**DOI:** 10.3390/ani15010040

**Published:** 2024-12-27

**Authors:** Weidong Chen, Yanshe Xie, Zhiqian Xu, Yijun Shang, Wenzheng Yang, Pengyao Wang, Zhenfang Wu, Gengyuan Cai, Linjun Hong

**Affiliations:** 1 State Key Laboratory of Swine and Poultry Breeding Industry, National Engineering Research Center for Breeding Swine Industry, Guangdong Provincial Key Laboratory of Agro-Animal Genomics and Molecular Breeding, College of Animal Science, South China Agricultural University, Guangzhou 510642, China; m18344306695@163.com (W.C.); 12217021@zju.edu.cn (Y.X.); qweguoqin123@163.com (Y.S.); y18672262036@stu.scau.edu.cn (W.Y.); pengyao@stu.scau.edu.cn (P.W.); wzf@scau.edu.cn (Z.W.); 2 College of Animal Science and Technology, Henan University of Science and Technology, Luoyang 471023, China; zqxu@haust.edu.cn; 3 Yunfu Subcenter of Guangdong Laboratory for Lingnan Modern Agriculture, Yunfu 527300, China; 4 Key Laboratory of South China Modern Biological Seed Industry, Ministry of Agriculture and Rural Affairs, Guangzhou 510520, China; 5 National Regional Gene Bank of Livestock and Poultry (Gene Bank of Guangdong Livestock and Poultry), Guangzhou 510642, China

**Keywords:** boar sperm, extracellular vesicles, miR-26a, biomolecular marker

## Abstract

Artificial insemination (AI), as an efficient assisted reproduction technology, can help the livestock industry to improve livestock and poultry breeds, optimize production performance and improve reproductive efficiency. AI technology has been widely used in pig production in China, but boar fertility affects the effectiveness of AI, and more and more studies have shown that there are significant differences in the fertility of boars with similar semen quality indicators. Therefore, this study aimed to identify biomarker molecules that indicate the level of boar fertility, which is important for improving the efficiency of AI. In this study, we collected 40 mL of ejaculates per boar used for extracellular vesicle (EV) characterization in 20 boars and identified 53 differentially expressed miRNAs by small RNA sequencing, of which 44 miRNAs were up-regulated in the high-fertility seminal EVs compared with low-fertility seminal EVs, and nine miRNAs were down-regulated. miR-26a was most significantly down-regulated in the high-fertility group compared to the low-fertility group, and it was hypothesized that this miRNA could be used as a biomolecular marker of semen reproductive performance. To further determine the effect of miR-26a on sperm function, we successfully established a miR-26a overexpression model and found that miR-26a reduced sperm viability, motility, acrosome integrity, plasma membrane integrity and ATP levels. Bioinformatics analysis and dual luciferase reporter analysis revealed that miR-26a directly targets High mobility group A1 (HMGA1). In conclusion, miR-26a can be used as a biomarker to identify high and low fertility in boar semen.

## Simple Summary

We selected high- and low-fertility boars and collected semen and found that miR-26a from boar seminal plasma extracellular vesicles could be useful as a biomarker to identify boar fertility. Experimentally, miR-26a was found to significantly inhibit sperm viability, motility, acrosome integrity, plasma membrane integrity and ATP levels, and may affect sperm function by targeting HMGA1.

## 1. Introduction

Artificial insemination (AI) is an assisted reproductive technique in which sperm is delivered into the female reproductive tract to achieve fertilization without sexual intercourse, in about 90% of swine used AI for breeding [1]. In addition, AI has found an increasingly wide utilization in the genetic improvement of pigs [2]. Therefore, selection for high-fertility boars is important in the genetic improvement of pigs. Over the past decades, semen analysis has been routinely performed as the first step in the assessment of male fertility [3]. However, the accuracy of conventional semen analysis (motility, morphology and sperm concentration) has always been controversial [4]. Currently, protein markers in spermatozoa were shown to discriminate boars with high fertility or low fertility [5,6], but more samples are required to validate their predictive utility. In addition, more biomarkers are needed for the integrative analysis to improve the predictive accuracy of semen analysis [7]. In this regard, development of novel methods for predicting male fertility of boar is urgent.

Semen is composed of sperm and seminal plasma, in which secretions derived from testes, epididymides and prostate, seminal vesicles, as well as the bulbourethral and periurethral glands accounted for 2–5%, 20–30%, 65–75% and 1%, respectively [8]. Seminal plasma (SP) contains a large number of growth factors and transcription factors, along with small molecules like amino acids, sugars and lipids, which provide adequate nutrition and an ideal environment when sperm travel through the female reproductive tract [9]. In addition, seminal plasma also plays key roles in sperm maturation, sperm capacitation and acrosome reaction, and thus fertilization [10]. Extracellular vesicles (EVs) are highly abundant in seminal plasma and control capacitation, acrosome reaction, and motility [11]. Interestingly, seminal EVs have been found to selectively deliver molecules into the female reproductive tract to promote fertilization [12].

EVs, comprising proteins and nucleotides, are nanoscale lipid bilayer-enclosed vesicles that are secreted by various cells [13]. Studies have shown that seminal fluid contains a large amount of EVs secreted by the testis, epididymis, prostate and seminal vesicle [14,15,16,17,18]. During spermatogenesis, the DNA in sperm are packaged, resulting in transcriptionally silent and translationally silent mature spermatozoa [19]. Therefore, the maturation and capacitation of sperm are controlled mainly through post-transcriptional modification and post-translational modification [20]. MicroRNAs (miRNAs) are small non-coding RNAs that affect mRNA stability and regulate translation at the post-transcriptional level by directly binding to the 3′Untranslated Region (3′UTR) site of target genes [21]. Previous research has shown that miRNAs are involved in several cellular processes, including proliferation, differentiation and apoptosis [22]. In addition, miRNAs are also involved in embryonic development [23], especially the male germline differentiation [24] and spermatogenesis [25].

EVs have a specific expression pattern of miRNAs that may reflect their cell origin [26]. In addition, the membrane of EVs could effectively protect enclosed miRNAs from the RNase [27]. Therefore, these EV-delivered miRNAs were used as non-invasive biomarkers for diseases [28]. Recently, Maria Barceló et al. found that seminal EV-delivered miRNAs can be used as a biomarker of azoospermia [29] and prostate cancer [30]. Xu et al. performed small RNA sequencing of EV-delivered small RNAs from boar seminal plasma and further illustrated the role of microRNAs (miRNAs) and PIWI-interacting RNAs (piRNAs) in sperm maturation, capacitation, acrosome reaction and fertility [31]. Vojtech et al. found that small non-coding RNAs (sncRNAs) were enriched in EVs and had potential regulatory functions in the female reproductive tract and fertility [32]. Above all, seminal EV-delivered miRNAs may serve as a useful biomarker to predict boar fertility.

In this study, EVs from seminal plasma were isolated and identified in high- and low-fertility boars. Subsequently, small RNA sequencing was performed to analyze differentially expressed miRNAs (DEM) in EVs. In addition, we predicted the target genes of DEMs and performed GO enrichment and KEGG pathway analysis to explore the potential biologic of DEMs. Additionally, the accuracy of sequencing data was validated using RT-qPCR. Furthermore, we investigated the impact of miR-26a on sperm viability, motility, acrosome integrity, plasma membrane integrity, reactive oxygen species, and ATP levels. The targeting relationship between miR-26a and High mobility group A1 (HMGA1) was validated by dual luciferase reporter assay. Moreover, the phosphorylation level of Adenosine 5′-monophosphate-activated protein kinase (AMPK) in sperm overexpressing miR-26a was detected to explore miR-26a as a potential biomarker for semen from high- and low-fertility boars. This study is of significant importance for understanding the molecular mechanisms of EV regulation in reproductive processes, and provides potential biomarkers for determining boar fertility. In frontline production, we may be able to identify boar semen reproductive performance by detecting miR-26a expression, thus greatly reducing the problem of higher production costs and lower returns due to substandard boar semen.

## 2. Materials and Methods

### 2.1. Ethics Statement

All the experiments involving animals were performed in adherence to guidelines by the Institutional Animal Care and Use Committee (IACUC) of South China Agricultural University (permit number: SYXK-2022-0136).

### 2.2. Semen Collection

We selected Large white pigs (6 boars in total, aged around 2 years old) with more than ten breeding records and corresponding sow farrowing records. We selected sows with roughly the same reproductive efficiency for breeding [33]. The boars were housed by the Wens Group (Yunfu, Guangdong Province, China), and the semen ejaculates were collected by the gloved-hand method. Sperm motility were evaluated by means of a Computer-aided Semen Analysis system (CASA, Barcelona, Spain). We screened high- and low-fertility boars based on the reproductive efficiency (ratio of total litter size of participating sows to the number of litters of sows mated to boars) of the participating sows (12.35 ± 0.25 and 10.88 ± 0.35, respectively) [34].

### 2.3. Evaluation of Semen Analysis

A quantity of 7 μL of the semen was placed into the prewarmed porcine sperm count four-chamber slide and viewed under a light microscope with a total magnification of 200× after being incubated with the control and treatment semen for 5 min at 37 °C.

### 2.4. Collection of Boar Seminal EVs of Different Fertility

EVs were isolated from semen as described previously with some modification. Briefly, we collected 40 mL of ejaculates per boar used for EVs characterization, and the semen was centrifuged at 800× *g* for 10 min to remove sperm. The supernatant was centrifuged at 16,000× *g* for 1 h at 4 °C to remove cellular impurities followed by ultra-centrifugation at 120,000× *g* for 1 h at 4 °C to obtain the EVs and EV-depleted supernatant. The EVs were washed twice with Tris-NaCl (30 mM Tris, 130 mM NaCl, pH 7.6) at 120,000× *g* for 1 h at 4 °C and further purified using Optiprep™ Density Gradient (ODG, Springfield, MO, USA).

Briefly, the EVs were re-suspended in 500 µL PBS and loaded to the top of a discontinuous OptiPrep™ density gradient prepared by diluting iodixanol (60% *w*/*v*; Sigma Aldrich, St. Louis, MI, USA, Cat. No. D1556) in PBS. The gradients were layered as 3 mL of OptiPrep™ solution 40%, 20%, 10% and 2.5 mL OptiPrep™ solution 5%. Samples were ultra-centrifuged at 120,000× *g* for 18 h (SW 41 Ti rotor, Beckman Coulter, Brea, CA, USA) after achieving the required tube fill volume with PBS, and each 1 mL ODG fractions was collected and re-suspended in PBS for a further 3 h ultra-centrifuged at 120,000× *g*. Finally, the EVs (ODG fraction) were collected for further analysis.

### 2.5. Transmission Electron Microscopy

The copper mesh was coated with 10 µL of EVs, which was incubated at room temperature for 10 min. The excess fluid around the copper mesh was blotted with filter paper, and 2% (*v*/*v*) uranyl acetate aqueous solution was added to the copper mesh. The grids were observed and photographed under a transmission electron microscope (Hillsboro, OR, USA) [35].

### 2.6. Nanoparticle Tracking Analysis

The concentration of EVs was diluted to 1 × 10^6^ to 1 × 10^9^ particles/mL with DPBS. A ZetaView PMX 110 (Meerbusch, Germany) equipped with a 488 nm laser was used to examine the sizes and quantities of particles isolated. The nanoparticle tracking analysis was utilized to scan 11 places at a frame rate of 30 frames per second for three cycles in order to analyze particle motion [36].

### 2.7. Western Blotting Analysis

EVs, sperm and EV-depleted supernatant were lysed in RIPA buffer containing 1% protease inhibitors, and protein concentrations were determined using the BCA kit. SDS-PAGE was used to separate protein samples, which were transferred to PVDF membranes activated with methanol. Subsequently, the membranes were blocked for 2 h at room temperature with 2% BSA. The membrane was washed three times with 1 × TBS. The washed membranes were incubated with primary antibodies (Table 1) for 12 h at 4 °C. The membrane was washed three times with 1 × TBST. Finally, the washed membranes were incubated with secondary antibodies. The signals of membranes were developed with an enhanced chemiluminescence (ECL, Beyotime, Shanghai, China, Cat. No. P0018S) reagent and captured by UVP system (Upland) [37].

### 2.8. RNA Extraction and Small RNA Sequencing

The total RNA of EVs was extracted by exoRNeasy Serum/Plasma Maxi Kit (Qiagen, Dusseldorf, Germany, Cat. No. 77023) according to the manufacturer’s instructions. For each sample, 20 ng of total RNA was used to generate Small-RNA cDNA library using NEBNext^®^ Multiplex Small RNA Library Prep Set for Illumina (NEB) according to the manufacturer’s instructions. The cDNA libraries were on the Illumina HiSeq 2500 platform (Illumina, San Diego, CA, USA) at Ribobio Co., Ltd. (Ribobio, Guangzhou, China) using 50 bp single-end reads.

Sperm were washed twice with DPBS at 2000× *g* for 5 min, followed by adding 1.5 mL trizol to lyse the sperm at room temperature for 10 min. Subsequently, 300 µL of chloroform was added and incubated on ice for 5 min, followed by centrifugation at 12,000× *g* for 15 min at 4 °C. The top layer of clear liquid was added to 700 µL of precooled isopropanol and 1 µL of glycogen and incubated on ice for 30 min to precipitate RNA, followed by centrifugation at 12,000× *g* for 30 min at 4 °C. After that, the precipitate was washed with 1 mL of 75% alcohol and centrifuged at 7500× *g* for 5 min at 4 °C. Finally, 20 µL DEPC was added to dissolve the precipitate [40].

### 2.9. Small RNA Sequencing Data Analysis

The raw reads were processed by filtering out reads without the 3′ adapter or the insert tag, with 5′ adapter contaminants, with a length less than 17 nt and containing poly A or T or G or C or N, and inferior-quality reads by FASTQC software (v0.11.8) to get clean reads. Mapping reads were obtained by mapping clean reads to the Bowtie reference genome. MiRDeep2 was used to identify known mature miRNA based on miRBase version 22. Next, databases of Rfam12.1 were used to identify rRNA, tRNA, snRNA and snoRNA by BLAST. The miRNA expression was calculated by RPM (Reads Per Million) values (RPM = (number of reads mapping to miRNA/number of reads in Clean data) × 10^6^). Differential expression between two sets of samples was calculated by the DESeq2 algorithm according to the criteria of |log2(Fold Change)| ≥ 1 and *p*-value < 0.05 [41].

### 2.10. Validation of miRNA Expression by RT-qPCR Analysis

The RNA samples from six sequenced donors (three high-fertility and three low-fertility boars) were extracted for the validation of miRNA expression. Six DEMs were randomly selected (ssc-miR-342, ssc-miR-26a, ssc-miR-210, ssc-miR-142-3p, ssc-miR-451, ssc-miR-223). Reverse transcription was performed with the Mir-X miRNA First-Strand Synthesis Kit (Takara, St. Louis, MO, USA, Cat. No. 638315), according to the manufacturer’s protocols. Subsequently, these cDNAs were validated by PowerUp™ SYBR™ Green Master Mix (Thermo Fisher, Waltham, MA, USA, Cat. No. A25742) on a Real-Time PCR System (Applied Biosystems, Foster City, CA, USA). The relative expression levels of miRNA were calculated using the 2^−ΔΔCt^ method [42].

### 2.11. Function Analysis of Differentially Expressed miRNAs

We used three established miRNA target prediction algorithms: miRanda [43], PITA [44], and RNAhybrid [45] to predict the target gene of DEMs. Only the mRNAs that were predicted by all three algorithms were selected for functional analysis. Subsequently, Gene Ontology (GO) enrichment and Kyoto Encyclopedia of Genes and Genomes (KEGG) pathway analysis of the target gene was performed with a David bioinformatics server [46].

### 2.12. Coincubation Experiment

To investigate the function of miR-26a in EVs, the transfection system was invented according to Ma’s patent [47]. The coincubation experiment included four experimental groups: a Mimic group (M), Mimic-negative control group (NC), Inhibitor group (I) and Inhibitor-negative control group (IC). For the M and NC transfection systems, 2.5 µL X-tremeGENE™ siRNA Transfection Reagent (St. Louis, MO, USA) and 50 µL Opti-MEM were premixed into solution A and incubated at room temperature for 5 min. A quantity of 2.5 µL miRNA Mimic/miRNA Mimic negative control and 50 µL Opti-MEM were premixed into solution B and incubated for 5 min. After that, solution A and B were mixed and incubated at room temperature for 20 min and added to 875 µL of semen. For the I and IC transfection systems, 5 µL X-tremeGENE™ siRNA Transfection Reagent and 50 µL Opti-MEM were premixed into solution C and incubated at room temperature for 5 min. A quantity of 5 µL miRNA Inhibitor/miRNA Inhibitor negative control and 50 µL Opti-MEM were premixed into solution D and incubated for 5 min. Subsequently, solutions C and D were mixed and incubated at room temperature for 20 min, and added to 870 µL of semen. Finally, the transfected semen was placed in an incubator at 17 °C for 6 h. All of the above reaction systems ended up with a volume of 1 mL.

### 2.13. Identification of Acrosome Integrity

The transfected semen was centrifuged at 2000× *g* for 3 min at room temperature, and the spermatozoa were resuspended in DPBS to adjust the sperm concentration to 5 × 10^6^ sperm/mL. Subsequently, 30 µL of semen was plated onto slides and air-dried in a ventilated cabinet. A quantity of 30 µL of absolute methanol was added for fixation at room temperature for 10 min, and washed three times with TBST. Afterwards, 30 µL FITC-PNA (100 μg/mL, dissolved in DPBS) was dropped to completely cover the semen, which was incubated in the dark at room temperature for 30 min, and washed three times with TBST. Finally, photographs were taken under a fluorescence microscope, and at least 200 spermatozoa were counted in each group [48].

### 2.14. Identification of Plasma Membrane Integrity

The transfected semen was centrifuged at 2000× *g* for 3 min at room temperature and resuspended in DPBS. Subsequently, 6-CFDA (10 µM, dissolved in DPBS) and Pi (10 µM, dissolved in DPBS) were added to a 1.5 mL centrifuge tube and heated in a 37 °C water bath for 10 min. Afterwards, 30 µL of semen was dropped onto a slide and covered with a cover slip. Finally, photographs were taken under a fluorescence microscope, and at least 200 spermatozoa were counted in each group [49].

### 2.15. Identification of ATP Levels

The transfected semen was centrifuged at 2000× *g* for 3 min at room temperature and resuspended in DPBS. A quantity of 100 µL of the resuspended semen was taken into a 96-well plate, and 100 µL of ATP assay reagent was added to each well. The plate was shaken at room temperature for 2 min, followed by a 10 min incubation at room temperature, and then analyzed using a multifunctional microplate reader [50].

### 2.16. Dual Luciferase Reporter Assay

We used PlTA, miRanda, and RNAhybrid to predict miR-26a target genes and chose HMGA1 for a luciferase reporter assay. To verify that HMGA1 interacted with miR-26a, the porcine HMGA1 gene’s 3′UTR binding site that binds to miR-26a was cloned into the pmirGLO vector (Promega, Madison, WI, USA), and the mutant plasmid was constructed by targeted mutagenesis. PK-15 cells were inoculated into cell culture plates at 1 × 10^4^ per well, and miR-26a mimics and dual-luciferase reporter plasmids (pmirGLO-HMGA1-WT, pmirGLO-HMGA1-MUT) were transfected into PK-15 cells using Lipofectamine 3000 (Waltham, MA, USA) transfection reagent. After 48 h of transfection, the treated cells were harvested and assessed for luciferase activity using the Dual-Luciferase Assay Kit (YENSEN, Shanghai, China) [51].

### 2.17. Statistical Analysis

All data were tested for normality and variance homogeneity prior to statistical analysis. Prism 8.0 (GraphPad) was used to perform data analysis, and a Student’s *t*-test was used to analyze significant differences between different groups. * *p* < 0.05 was considered statistically significant; ** *p* < 0.01 was considered especially significant.

## 3. Results

### 3.1. Characterization of Seminal EVs

**Figure 1:**
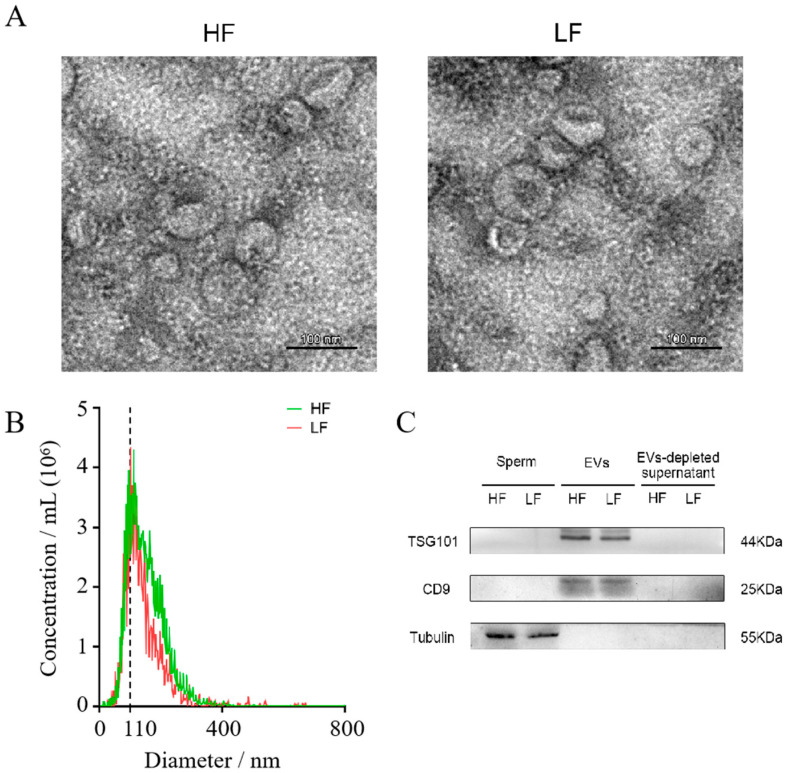
Characterization of high-fertility and low-fertility seminal EVs. (**A**) Transmission electron micrographs analysis revealed that seminal EVs possess typical cup-shaped vesicle structures. Scale bar = 100 nm. (**B**) Nanoparticle tracking analysis of particle size distribution profiles from high-fertility and low-fertility seminal EVs; most particle sizes range from 50 to 300 nm, consistent with the characteristics of EVs. (**C**) Western blotting detected EV protein markers TSG101 and CD9 in the seminal EVs from high-fertility and low-fertility boars. The cytoplasmic marker was only detected in the sperm. HF, high fertility; LF, low fertility.

Seminal EVs were collected from boars with high fertility and low fertility. The morphology of seminal EVs were characterized by TEM and presented cup-shaped vesicle structures (Figure 1A). NTA analysis revealed that most seminal EVs had a diameter of between 50 and 300 nm and a primary peak having a particle size of about 110 nm (Figure 1B). Western blotting analysis revealed that seminal EVs were positive for EV protein markers (TSG101 and CD9), and negative for cytoplasmic contaminants (Figure 1C).

### 3.2. Overview of the Sequencing Data

**Figure 2:**
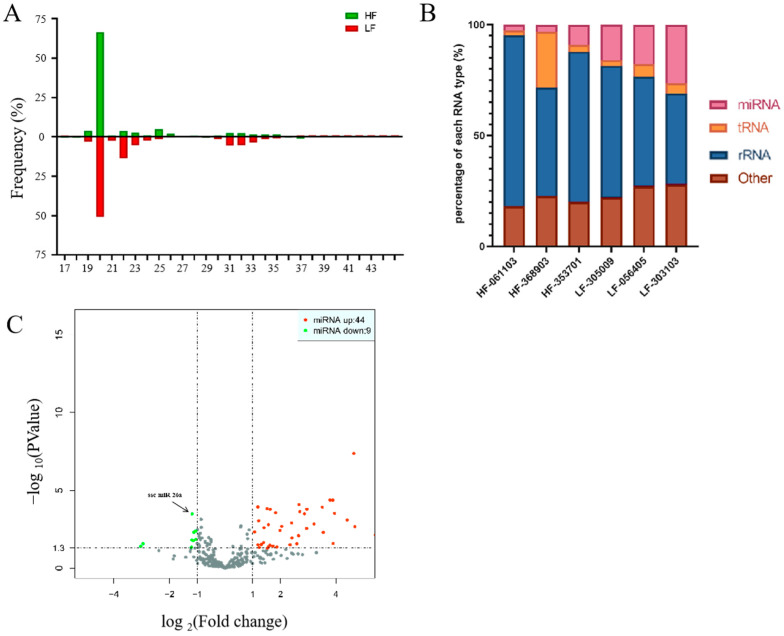
Overview of the sequence data. (**A**) Length distribution of clean reads from two libraries. The length distribution peaked at 20 nt. HF, high fertility; LF, low fertility. (**B**) Relative abundance of different classes of RNA in high-fertility and low-fertility seminal EVs. (**C**) Volcano plots of differentially expressed miRNAs. The *X*-axis denotes the value of log2 (Fold change) and the *Y*-axis refers to the *p* value (−log 10).

Six small RNA libraries were generated from high-fertility (*n* = 3) and low-fertility (*n* = 3) seminal EVs. On average, 66.59% and 50.89% of the total clean reads comprised 20 nucleotides (nt) in the high-fertility and low-fertility libraries, respectively (Figure 2A). Of these reads, 792,290 (5.15%) and 3,714,734 (20.19%) were identified as the miRNA. The rest of the sequences were found to be other types of RNA, including tRNA, rRNA, snRNA, snoRNA, YRNA and others (Figure 2B). There were 53 DEMs with |log2(Fold Change)| ≥ 1 and *p*-value < 0.05 identified between the high-fertility and low-fertility groups, of which 44 miRNAs were up-regulated in the high-fertility seminal EVs compared with low-fertility seminal EVs, and nine miRNAs were down-regulated (Figure 2C). The down-regulation of miR-26a was the most significant. Interestingly, miR-26a was reported to be closely associated with sperm motility. Therefore, we selected miR-26a as a candidate gene.

### 3.3. RT-qPCR Validation of Sequencing Data

**Figure 3:**
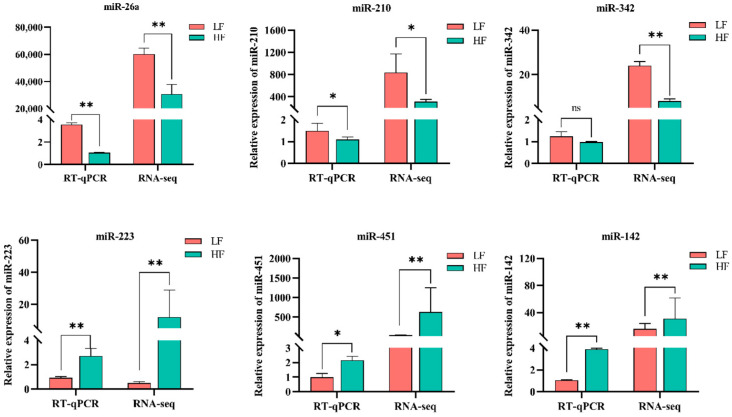
Validation of the expression of miRNAs by using RT-qPCR. The relative expression level of DEMs was consistent with the sequencing data. HF, high fertility; LF, low fertility. * *p* < 0.05 was considered statistically significant; ** *p* < 0.01 was considered especially significant. Values are expressed as mean ± SD (*n* = 5).

Six DEMs (miR-342, miR-26a, miR-210, miR-142-3p, miR-451, miR-223) were randomly selected to validate sequencing data using RT-qPCR of total RNA isolated from seminal EVs from ten un-sequenced donors. Results showed that the relative expression level of selected miRNAs was consistent with the sequencing data (Figure 3).

### 3.4. Functional Annotations of Differentially Expressed miRNAs

**Figure 4:**
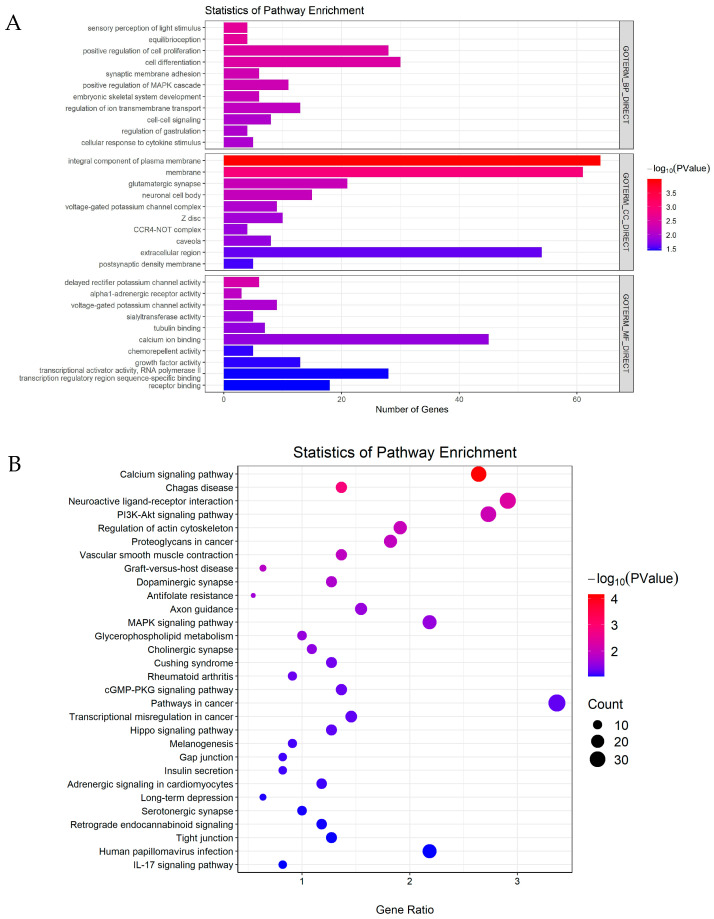
Functional annotations of differentially expressed miRNAs. (**A**) GO analysis of the target predicted genes of DEMs. (**B**) KEGG pathway analysis of the target predicted genes of DEMs.

To explore the potential biological potential of DEMs, GO enrichment and KEGG pathway analysis were performed on the target genes of DEMs. GO enrichment analysis showed that these target genes were mainly involved in biological processes such as positive regulation of cell proliferation, cell differentiation, positive regulation of MAPK cascade, regulation of ion transmembrane transport, cell–cell signaling, and cellular response to cytokine stimulus, which are related to sperm function, endometrial receptivity and embryo implantation. For molecular functions, these target genes were annotated as calcium ion-binding, which are essential for sperm capacitation (Figure 4A). KEGG pathway analysis of these target genes showed a significant enrichment in the calcium signaling pathway, regulation of actin cytoskeleton, glycerophospholipid metabolism and the cGMP-PKG signaling pathway, which are essential for sperm function. Additionally, the pathways associated with embryo implantation (MAPK signaling pathway, hippo signaling pathway, gap junction and tight junction) and immune (IL-17 signaling pathway) were also enriched. In particular, the PI3K-Akt signaling pathway and MAPK signaling pathway were crucial for both sperm function and embryo implantation (Figure 4B).

### 3.5. ssc-miR-26a Was Transported to Sperm by SPEVs

**Figure 5:**
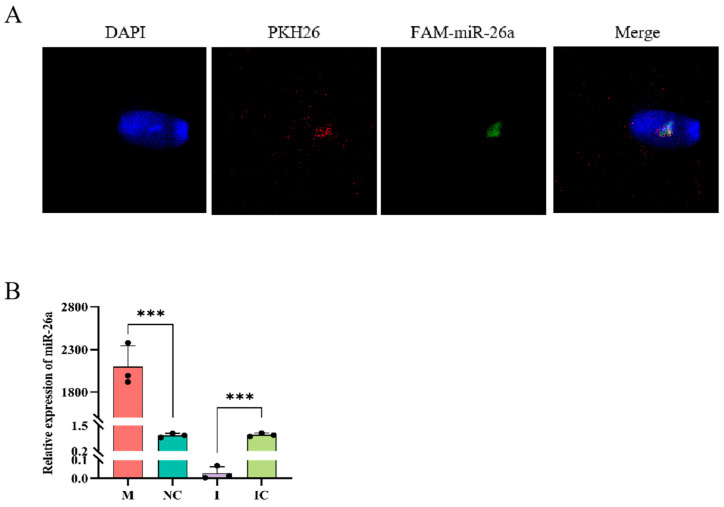
miR-26a could be transported to sperm via seminal plasma extracellular vesicles (SPEVs). (**A**) Internalization of SPEVs carrying miR-26a by sperm was observed by confocal microscopy. (**B**) RT-qPCR detected significant up-regulation of miR-26a expression in sperm after co-incubation with EVs. miR-26a mimic (M), miR-26a negative control (NC), miR-26a inhibitor (I), miR-26a inhibitor negative control (IC). The following experiments all follow this grouping. Values are expressed as mean ± SD (*n* = 3). *** *p* < 0.001.

To investigate whether miR-26a could enter sperm through the SPEVs, we labeled EVs with PKH26 and transfected FAM-miR-26a into EVs by transfection reagent, and incubated the treated EVs with spermatozoa for 24 h. The results are shown in Figure 5A,B, which shows that sperm ingests extracellular vesicles to the spermic head, and RT-qPCR results showed a significant increase in the miR-26a expression level in the miR-26a mimic group (*p* < 0.001). In contrast, the expression level of miR-26a decreased significantly in the miR-26a inhibitor group (*p* < 0.001). Taken together, we successfully constructed an miR-26a overexpression model, and miR-26a could enter sperm through EVs.

### 3.6. miR-26a Affects Sperm Motility

**Figure 6:**
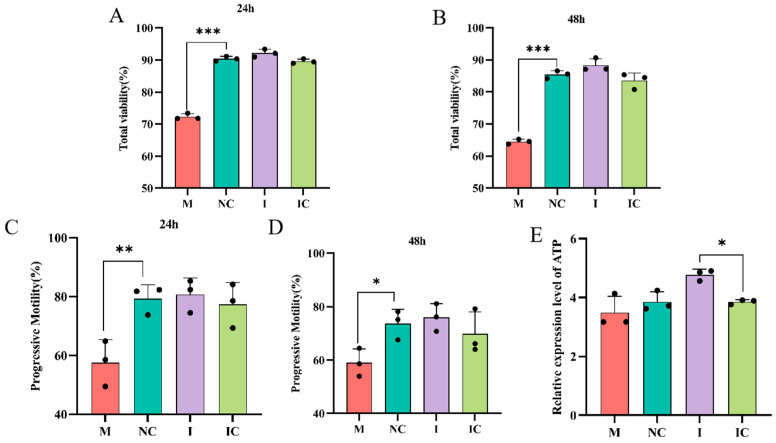
Effect of mIR-26a on sperm in vitro. (**A**,**B**) Detection of sperm viability by CASA at 24 h and 48 h after transfection with miR-26a. (**C**,**D**) Detection of sperm motility by CASA at 24 h and 48 h after transfection with miR-26a. (**E**) ATP levels of transfected sperm were detected using an ATP assay kit, and the results were counted by a multifunctional enzyme labeling instrument. Values are expressed as mean ± SD (*n* = 5). * *p* < 0.05, ** *p* < 0.01, *** *p* < 0.001.

To investigate the biological function of miR-26a in spermic motility, we detected sperm viability and motility at 24 h and 48 h after transfection by CASA. The results showed that miR-26a significantly decreased sperm viability and motility compared with the control group from 24 h to 48 h (Figure 6A–D).

Furthermore, we assessed the levels of ATP in sperm incubated with SPEVs with miR-26a. We observed that miR-26a induced a decrease in spermic ATP levels. As shown in Figure 6E, miR-26a significantly decreased the levels of ATP in sperm compared with the control group (*p* < 0.05) at 24 h, which further confirmed that the number of apoptotic sperm increased after the increased in miR-26a expression.

### 3.7. Phosphorylation of AMPK

**Figure 7:**
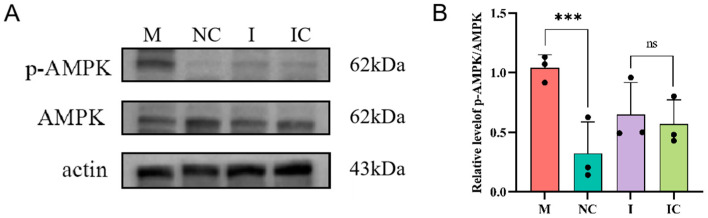
miR-26a may affect sperm function through the AMPK pathway. (**A**) Western blot assays were used to evaluate the total protein levels of the phosphorylation levels of AMPK in sperm incubated with miR-26a mimic (M), miR-26a negative control (NC), miR-26a inhibitor (I) and miR-26a inhibitor negative control (IC). (**B**) Values are expressed as mean ± SD (*n* = 3),*** *p* < 0.001; ns: *p* > 0.05.

AMP-activated protein kinase (AMPK) acts as a key physiological energy sensor and may be activated when ATP is reduced. We detected phosphorylation of AMPK by Western blot analysis. The results showed that the expression of AMPK phosphorylation increased in the miR-26a mimic group (Figure 7A,B). In conclusion, miR-26a may have effects on sperm function through activation of AMPK phosphorylation.

### 3.8. Effects of miR-26a on Sperm Acrosome Integrity

**Figure 8:**
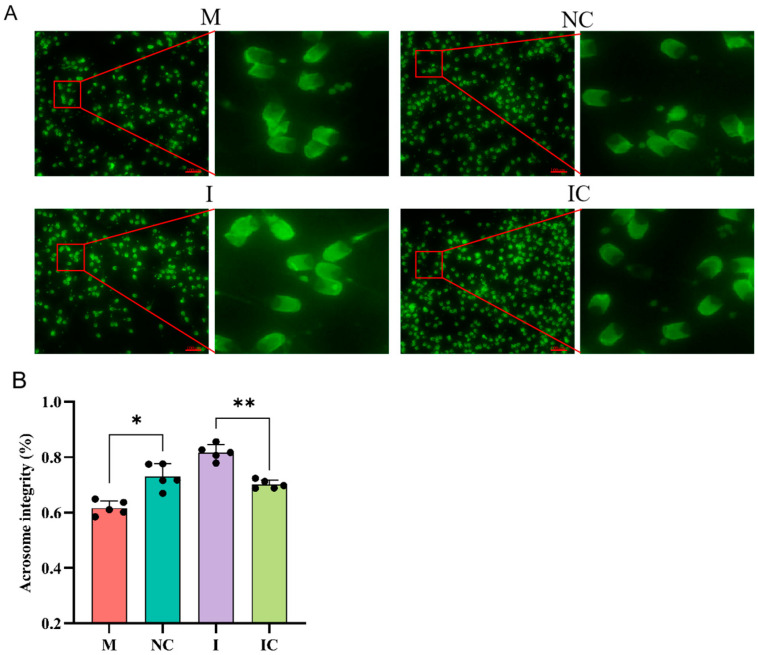
Detection of sperm acrosome integrity by FITC-PNA. (**A**) Sperm acrosome integrity was assessed by fluorescence microscopy, and sunken, non-smooth acrosomes were considered incomplete. Scale bars: 100 µm. Magnification: 400×. (**B**) Values are expressed as mean ± SD (*n* = 5), * *p* < 0.05, ** *p* < 0.01.

To determine the effect of miR-26a on sperm function, we detected acrosomal integrity of sperm by FITC-PNA. The results are shown in Figure 8A,B, which show that the acrosome integrity rate was significantly decreased in the miR-26a mimic group compared to the miR-26a-negative control group (*p* < 0.05). In contrast, the acrosome integrity rate was significantly increased in the miR-26a inhibitor group compared to the miR-26a inhibitor-negative control group (*p* < 0.01). In summary, miR-26a disrupts sperm acrosome structure and reduces sperm acrosome integrity.

### 3.9. Effects of miR-26a on Sperm Plasma Membrane Integrity

**Figure 9:**
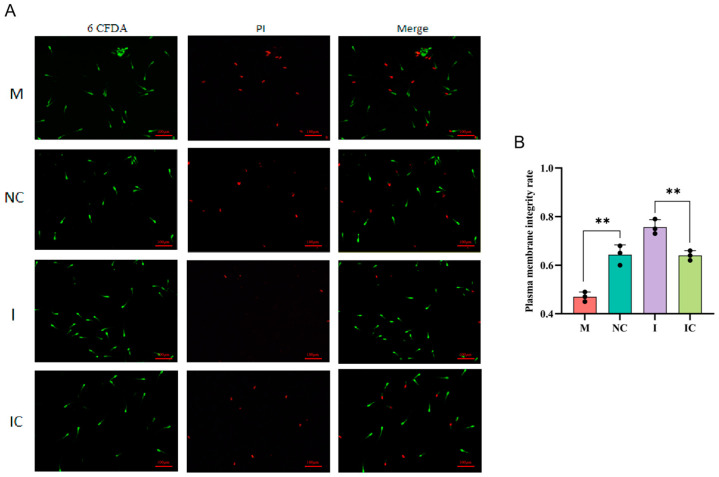
Detection of sperm plasma membrane integrity by dual fluorescent labelling method. (**A**) Sperm plasma membrane was assessed by fluorescence microscopy. Scale bars: 100 µm. Magnification: 200×. (**B**) Values are expressed as mean ± SD (*n* = 3), ** *p* < 0.01.

To further determine the effect of miR-26a on sperm function, we detected the sperm plasma membrane by dual fluorescent labelling (6-CFDA/Pi). The results are shown in Figure 9A,B, which show that sperm plasma membrane integrity was significantly decreased in the miR-26a mimic group compared to the miR-26a-negative control group (*p* < 0.01). In contrast, sperm plasma membrane integrity was significantly increased in the miR-26a inhibitor group compared to the miR-26a-inhibitor negative control group (*p* < 0.01). In summary, miR-26a suppresses sperm plasma membrane integrity.

### 3.10. HMGA1 Is a Target Gene of miR-26a

**Figure 10:**
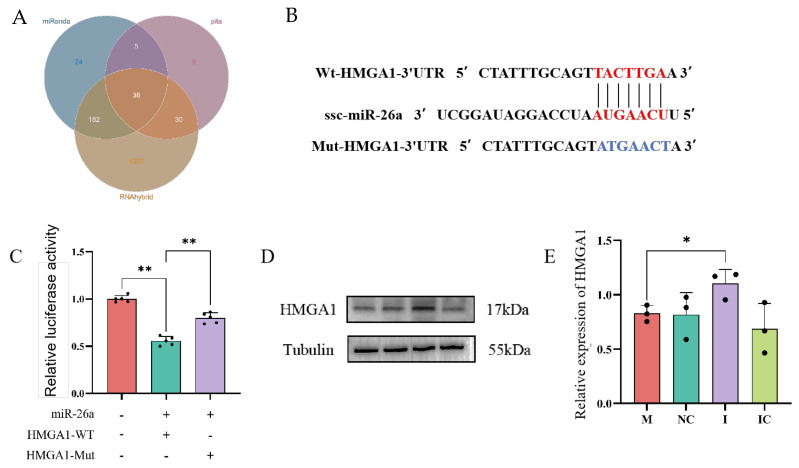
miR-26a targets and regulates the HMGA1 gene in sperm. (**A**) The target genes of miR-26a as predicted by miRanda, RNAhybrid and Pita databases. (**B**) Prediction of binding sites and mutation sites of miR-26a to the 3′UTR of porcine HMGA1 gene. (**C**) Relative active expression of luciferase after co-transfection of miR-26a mimics with wild-type (WT) HMGA1 3′UTR and mutant (Mut) HMGA1 3′UTR, respectively. (**D**,**E**) Protein blotting showed a significant increase in HMGA1 protein expression levels in the transfected miR-26a inhibitor group compared to the miR-26a mimic group. Values are expressed as mean ± SD (*n* = 3), * *p* < 0.05, ** *p* < 0.01.

To investigate the regulatory mechanism of miR-26a in sperm, a total of 1585 target genes were predicted using miRanda (Blue), RNAhybrid (Orange) and Pita (Pink), of which, 38 target genes were identified by the intersection of results of the three online tools (Figure 10A). Among these 38 target genes, HMGA1 was reported as playing an indelible role in requiring for normal sperm development [36]. Therefore, we selected HMGA1 for subsequent validation. The 3′UTRs of HMGA1 was found to contain a miR-26a target-binding site (Figure 10B). Wild-type or mutant HMGA1-3′UTR dual luciferase reporter plasmids were constructed, and Figure 10C shows the results that dual luciferase reporter assays identified a significant decrease in dual luciferase activity after co-transfection of miR-26a with WT-HMGA1-3′UTR (*p* < 0.01), while co-transfection with Mut-HMGA1-3′UTR showed a significant increase in dual luciferase activity compared to the WT group (*p* < 0.01). Furthermore, Western blot analysis revealed a significant increase in HMGA1 protein expression in the miR-26a inhibitor group (Figure 10D,E). In conclusion, miR-26a could direct target HMGA1.

## 4. Discussion

In pig breeding, the accurate prediction of boar fertility is of great importance in order to significantly improve the insemination rate of artificial insemination, as has been confirmed by studies [52]. However, there are numerous limitations in the current analysis method, which is unable to accurately determine the reproductive performance of boars by sperm viability and morphology alone [53]. It is therefore of great urgency to improve the method.

EVs are a prevalent component of boar semen and have been demonstrated to play a pivotal role in numerous crucial aspects of reproductive physiology. These include sperm maturation [54], sperm motility [55], sperm capacitation [56], endometrial receptivity [57] and uterine immunomodulation [58]. A substantial body of research has substantiated the pivotal role of semen EVs in the intricate and vulnerable process of fertilization [59]. Moreover, the substances they convey have been identified as promising biomarkers of fertility. Following their release from the testis, spermatozoa enter a special state of transcriptional and translational repression, which implies that the subsequent maintenance or alteration of sperm function is largely dependent on post-translational modifications [60]. Among these, miRNAs represent a crucial class of modifiers. Recent studies have demonstrated the intimate relationship between miRNAs and reproductive processes, including sperm motility, sperm apoptosis and embryo implantation [61]. In this study, we screened sows with more than 10 matings and ensured that the pregnancy status of these sows was consistent. The pregnancy data were then used to identify high- and low-fertility boars. To further elucidate the miRNA expression profile associated with boar fertility, small RNAs were sequenced, and a total of 53 differentially expressed miRNAs (DEMs) were detected between the high- and low-fertility groups. Among these miRNAs, ssc-miR-26a expression showed a most significant decrease and increase in seminal EVs of high-fertility boars. Furthermore, evidence indicates that ssc-miR-26a is capable of precisely regulating glucose metabolism by targeting the pyruvate dehydrogenase complex (PDHX), which ultimately exerts a profound effect on boar spermatozoa viability [62]. Additionally, miR-26a has been demonstrated to negatively regulate the affinity of porcine trophoblast cells for laminin [63], and to induce apoptosis in goat stromal cells [64]. Furthermore, miR-26a has been demonstrated to suppress the expression of immune-related genes (e.g., IL-6 and IL-7) in mice [65], which may impede the establishment of an optimal uterine environment for fertilization and even implantation. In conclusion, the evidence suggests that the elevated expression of ssc-miR-26a is likely to result in a reduction in fertility in boars. This conclusion is supported by the reproductive performance data obtained. We hope to continue to analyze the correlation studies between differentially expressed miRNAs and sperm function next, which will greatly improve the reliability of miR-26a as a marker for identifying boar reproductive performance.

On the other hand, HMGA1 is a non-histone chromatin structural protein that does not exhibit transcriptional activity [66]. A substantial body of evidence from numerous studies has confirmed that HMGA1 plays a regulatory role in genes associated with tumors in a number of different systems, including the reproductive [67], digestive [68], urinary [69] and haematopoietic systems [70]. In this study, we employed three online tools, miRanda, RNAhybrid and Pita, to analyze HMGA1 as a potential target gene of miR-26a. Furthermore, we conducted triple validation with dual luciferase reporter genes, RT-qPCR and Western blotting to confirm the existence of a targeting relationship. Unfortunately, it is challenging to perform in vitro replication experiments by inhibiting HMGA1 mRNA due to the lower translation level of spermatozoa compared to somatic cells.

In this study, a high expression of miR-26a was observed to significantly inhibit several key sperm parameters, including viability, motility, acrosome integrity, plasma membrane integrity and ATP levels. Additionally, there was evidence to suggest that miR-26a may activate AMPK phosphorylation. It can therefore be concluded that miR-26a has the potential to serve as an effective marker for boar fertility. The successful application of miRNAs as biomarkers in breeding practices could have a profound impact on the pig industry. It has the potential to significantly reduce the cost of frontline production while simultaneously enhancing reproductive efficiency in sows, increasing the number of piglets per litter, and improving the health and survival rate of piglets. This could serve as a catalyst for the economic benefits and sustainable development of the entire pig industry.

## 5. Conclusions

miR-26a may regulate sperm function by targeting HMGA1, with significant inhibitory effects on sperm viability and motility, and acrosome, plasma membrane and ATP levels. Furthermore, miR-26a may be utilized as a biomarker to identify high and low fertility in boar semen.

## Figures and Tables

**Table 1:** Antibody basic information.

Antibody Name	Product Number	Company
Phospho-AMPKalpha	50081S	CST (Danvers, MA, USA)
AMPKalpha	5831T	CST (Danvers, MA, USA)
Anti-HMGA1	ab129153	Abcam (Cambridge, UK)
TSG101	381538	ZEN-BIOSCIENCE (Chengdu, China)
CD9	20597-1-AP	Proteintech (Rosemont, IL, USA)
Anti-beta Tubulin	GB11017-100 [38]	Servicebio (Wuhan, China)
Anti-Actin	GB113225-100 [39]	Servicebio (Wuhan, China)

## Data Availability

The raw sequence data reported in this paper have been deposited in the Genome Sequence Archive [71] in the National Genomics Data Center [72], China National Center for Bioinformation/Beijing Institute of Genomics, Chinese Academy of Sciences (GSA: CRA019897), which are publicly accessible at https://ngdc.cncb.ac.cn/gsa (accessed on 1 December 2024).
